# Meeting the Challenges of Myocarditis: New Opportunities for Prevention, Detection, and Intervention—A Report from the 2021 National Heart, Lung, and Blood Institute Workshop

**DOI:** 10.3390/jcm11195721

**Published:** 2022-09-27

**Authors:** Daniela Čiháková, Yang Shi, Bishow Adhikari, W. Patricia Bandettini, Madeleine W. Cunningham, Narasimhan Danthi, Matthias G. Friedrich, Peter Liu, Lisa Schwartz Longacre, Douglas L. Mann, Filip K. Swirski, W. H. Wilson Tang, Guofei Zhou, Leslie T. Cooper, Jr.

**Affiliations:** 1 Department of Pathology, Johns Hopkins University School of Medicine, Baltimore, MD 21205, USA; 2 W. Harry Feinstone Department of Molecular Microbiology and Immunology, Johns Hopkins University Bloomberg School of Public Health, Baltimore, MD 21205, USA; 3 Division of Cardiovascular Sciences, National Heart, Lung, and Blood Institute, National Institutes of Health, Bethesda, MD 20892, USA; 4 Department of Microbiology and Immunology, University of Oklahoma Health Sciences Center, Oklahoma City, OK 73104, USA; 5 Department of Diagnostic Radiology, McGill University Health Centre, Montreal, QC H4A 3J1, Canada; 6 Department of Medicine and Cellular Molecular Medicine, University of Ottawa, Ottawa, ON K1Y 4W7, Canada; 7 Center for Cardiovascular Research, Washington University School of Medicine, St. Louis, MO 63110, USA; 8 Cardiovascular Research Institute and the Department of Medicine, Icahn School of Medicine at Mount Sinai, New York, NY 10029, USA; 9 Department of Cardiovascular Medicine, Heart, Vascular and Thoracic Institute, Cleveland Clinic, Cleveland, OH 44195, USA; 10 Division of Lung Diseases, National Heart, Lung, and Blood Institute, National Institutes of Health, Bethesda, MD 20892, USA; 11 Department of Cardiovascular Medicine, Mayo Clinic, Jacksonville, FL 32224, USA

**Keywords:** myocarditis, dilated cardiomyopathy, viral myocarditis, cytokines, macrophages, lymphocytes, cardiac magnetic resonance, heart biopsy, heart failure, NHLBI workshop in myocarditis

## Abstract

The National Heart, Lung, and Blood Institute (NHLBI) convened a workshop of international experts to discuss new research opportunities for the prevention, detection, and intervention of myocarditis in May 2021. These experts reviewed the current state of science and identified key gaps and opportunities in basic, diagnostic, translational, and therapeutic frontiers to guide future research in myocarditis. In addition to addressing community-acquired myocarditis, the workshop also focused on emerging causes of myocarditis including immune checkpoint inhibitors and SARS-CoV-2 related myocardial injuries and considered the use of systems biology and artificial intelligence methodologies to define workflows to identify novel mechanisms of disease and new therapeutic targets. A new priority is the investigation of the relationship between social determinants of health (SDoH), including race and economic status, and inflammatory response and outcomes in myocarditis. The result is a proposal for the reclassification of myocarditis that integrates the latest knowledge of immunological pathogenesis to refine estimates of prognosis and target pathway-specific treatments.

## 1. Introduction

The World Health Organization (WHO) defines myocarditis as an inflammatory disease of the myocardium and is diagnosed by established histological, immunological, and immunohistochemical criteria [1]. However, the established criteria have low sensitivity and poor prognostic value, and they do not integrate current understanding of immunological homeostatic and regulatory functions with disease phenotypes. The plasticity of immune and stromal cells and their cell–cell interactions in three dimensions can now be more accurately quantified in myocardial health and disease states. These insights can be integrated with genomic patterns through systems biology and artificial intelligence algorithms to identify new immunophenotypes and therapeutic targets. From a clinical presentation perspective, the 2013 European Society of Cardiology (ESC) position statement on myocarditis describes an acute coronary syndrome in the absence of angiographic obstructive epicardial coronary artery disease often associated with chest pain, a respiratory or gastrointestinal infection, and electrocardiographic changes, as well as possible right and/or left ventricular dysfunction and elevated troponin levels. Advances in tissue imaging with echocardiography, magnetic resonance imaging, and positron emission tomography have led to higher rates of suspected and diagnosed cases of myocarditis and a need to integrate multimodality imaging tools into refined prognostic algorithms. At the same time, highly translational animal models and human studies are providing new insights into molecular mechanisms that can be leveraged to create novel imaging markers. These advances in basic and clinical science are occurring within a framework of social disparities, which also affects clinical outcomes.

The workshop, “Meeting the Challenges of Myocarditis: New Opportunities for Prevention, Detection, and Intervention Workshop,” held by the NHLBI on May 3, 4 and 6, 2021 discussed major advances in myocarditis research and identified key knowledge gaps in diagnostic, therapeutic, basic science, and translational frontiers for myocarditis. Here, we report on the challenges and opportunities discussed during the workshop, elaborating on a previously published Executive Summary [2].

## 2. Etiologies of Myocarditis

Most cases of myocarditis have been attributed to viral infections. The incidence of myocarditis is estimated to be 6.1 in men and 4.4 in women per 100,000 persons between ages 35 and 39 [3]. The mechanism of cardiac damage might be in part driven by the type of virus that infects the myocardium. Enteroviruses, herpesviruses, and adenoviruses are commonly found in myocarditis biopsies [4]. Other viruses, such as parvovirus B19. can persist in the cardiomyocytes with an unknown consequence to myocarditis pathogenesis [5]. It is important to distinguish between myocarditis caused by viral infection or associated with a virus presence and further enhance our knowledge of viral presence and cause of myocarditis. This includes the use of polymerase chain reaction (PCR) to detect viral genome in the heart since this technique cannot distinguish virus in the tissue from a virus in the blood without simultaneous testing of peripheral blood. Some viruses are also ubiquitous and present in a fraction of healthy hearts at autopsy. SARS-CoV-2 has been also shown to induce an acute cardiac injury, reflected by an elevated serum level of cardiac troponin, in severe COVID-19 patients [6]. The presence of SARS-CoV-2 in the heart is associated with increased myeloid cells density [7,8]. Cardiac injury could be also caused indirectly by cytokines or immune dysregulation. However, classic lymphocytic myocarditis is rarely found in COVID-19 autopsies [9]. Alternative mechanisms of injury such as platelet-rich microthrombi and direct proarrhythmic effects of viral proteins have been reported in COVID-19 decedents [10].

Other less common causes of myocarditis are parasitic, bacterial, and fungi infections, as well as toxins and hypersensitivity reactions. Immune checkpoint inhibitors (ICI) myocarditis occurs in 0.3 to 1% of ICI- treated cancer patients with up to a 50% reported mortality [11,12]. Although myocarditis after vaccination for influenza, hepatitis B, and varicella [13,14,15,16] is rare, mRNA COVID-19 vaccines (mRNA-1273 [Moderna], and BNT162b2 [Pfizer]) have been reported to trigger myocarditis with an estimated risk of 3.8 per 100,000 in males and 0.5 in 100,000 females [17,18]. Young males between 16 and 29 years of age had the highest incidence of post-mRNA vaccine myocarditis at 10.7 cases per 100,000 [19]. Histologically, the post-COVID-19-vaccine myocarditis is characterized by cardiomyocyte damage and mixed inflammatory infiltration with scattered eosinophils [19,20]. The myocarditis incidence is usually observed several days after the second vaccination (or after the first vaccination in people with a previous COVID-19 infection). Although 76% or more of these cases have little to no impairment of left ventricle systolic function and patients recover within days, understanding the mechanism of this injury is of great interest in the COVID-19 pandemic [19].

Autoimmune inflammation causes giant cell myocarditis and, in some cases, eosinophilic myocarditis [21,22,23,24,25,26]. Myocarditis is associated with high morbidity when associated with systemic autoimmune diseases, such as Sjögren’s disease, systemic lupus erythematous, and vasculitis [27,28]. In addition, sarcoidosis, an inflammatory granulomatous disease, can affect the heart [29]. The precise mechanism by which acute viral infection progresses to chronic autoimmune myocarditis is incompletely characterized, but may involve molecular mimicry between viral and cardiac antigens as well as potential bystander effects during antigen presentation [30,31]. Results from a mouse model of Coxsackievirus-induced myocarditis suggests that autoimmune inflammation develops as a sequela of a virus infection with anti-cardiac antibodies induced by the viral infection and prolonged myocarditis after viral clearance in susceptible mice strains.

**Gaps and Opportunities in Myocarditis Etiology**:

- Although viruses are the most common cause of myocarditis, RT-PCR and genomic sequencing testing for viruses in biopsies may not correlate with active infection.
- Therefore, greater understanding is needed of the mechanisms of specific viruses for myocarditis pathogenesis. Specifically, research is needed to understand the factors which determine when acute viral myocarditis progresses to an autoimmune cardiomyopathy in humans.
- Understanding the mechanism of mRNA vaccine-induced myocarditis is important for the use of this technology to prevent COVID-19 and to understand the risks of other vaccines based on the same technology.
- It is critical to understand the broader scope of pathogenesis of inflammatory myocardial diseases in general is critical.

## 3. Pathogenesis of Myocarditis

The pathogenic process of myocarditis is often driven by activated immune cells, cytokines, and chemokines [32]. Identifying key mechanisms that can either propagate or protect against myocarditis still requires a more granular understanding of the role of relevant immune cells. The heart contains subsets of tissue-resident macrophages with different ontologies that can be repopulated through either the recruitment of circulating monocytes or local self-renewal. During the disease process, monocytes infiltrate the heart and produce macrophages with diverse and seemingly opposite functions. Macrophages help maintain immune homeostasis, but they can also contribute to pathologic inflammation. The interplay between cardiac stromal cells and monocytes has been described recently [33]. The types of monocytes infiltrating the heart and the in situ differentiation of these cells are driving the fibrotic process in mouse models [34,35]. Advances in myocardial biology and immunology will likely pave the way for newer insights into the roles of the various myeloid cells in myocarditis. For example, CCR^2+^Ly6C^hi^ inflammatory macrophages were shown to drive post-myocarditis fibrosis and development of dilated cardiomyopathy (DCM) [35]. Derived from extra-embryonic hematopoietic progenitors, cardiac resident immune cells produce growth factors and are key regulators of coronary development, angiogenesis, and cardiac tissue repair in the diseased heart [36,37,38]. They may drive inflammatory responses by generating cytokines, oxidative species, and chemokines that trigger neutrophil and monocyte infiltration [36,37,38].

The recruitment of immune cells to the inflamed or injured myocardium is dynamic as well as cell-type specific and context dependent. Genetic, chimeric, and computational tools have shown that under homeostatic conditions, a diverse population of macrophage and dendritic cell subpopulations gain temporary residency within the injured heart, proliferate within cardiac tissue transiently, and are continually replaced by circulating progenitors [39,40,41]. We have some evidence about divergent roles of subtypes of monocytes in myocarditis, but the full relevance of such transcriptional plasticity of monocytes in the context of myocarditis is still unclear [33]. In addition, environmental influences can promote inflammatory responses, especially in genetically susceptible individuals, as recently demonstrated in the activation of cardiac myosin-specific T-helper cells in the intestine by a mimic peptide from a common commensal *Bacteroides* species in patients with particular HLADQA1*/B1* alleles [42].

**Figure 1:**
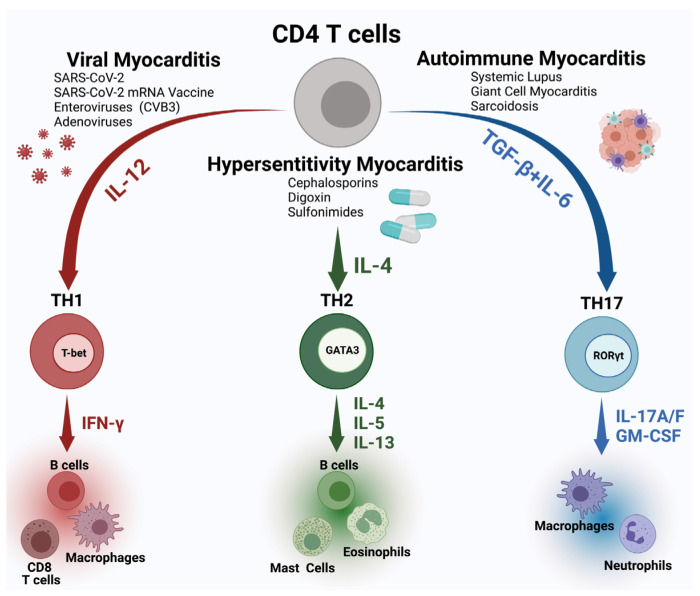
**T helper cell pathways leading to three subtypes of myocarditis.** Viral myocarditis activating Th1 pathway driven by IL-12 induces IFNγ production and CD8^+^ T cells as the main effector cells. Hypersensitivity myocarditis is induced by Th2 pathway driven by cytokines (IL-4, IL5, and IL13), mast cells, eosinophils, and B cells. Autoimmune myocarditis is driven by the Th17 pathway, with the IL17A/ F, and GM-CSF as the main cytokines inducing neutrophil and inflammatory monocyte responses.

Therefore, understanding the regulatory switch between cardiac inflammatory and reparative responses to viral infection is critical yet remains elusive. Long thought to be primarily inflammatory, neutrophils can also regulate inflammation and warrant further study. Although T cell-mediated damage occurs via CD8+ T cells that recognize major histocompatibility complex (MHC) I on infected cardiomyocytes, immune equilibrium is mediated by various T cell subsets. While significant strides in understanding of how Th1, Th2, Th17, T regs, and other T cell subtypes respond to viral infection and modulate inflammation and fibrosis have been made, more work is still needed (Figure 1) [34,43,44,45,46,47,48,49,50,51]. The etiology of the myocarditis dictates the immune profile found in the myocardium. Viral myocarditis is driven by a Th1 response, and the histological profile is mostly lymphocytic with inflammatory monocytes being the predominant cell type in the myocardium. The Th17 response plays a role in autoimmune myocarditis and this type of response drives the post-myocarditis dilated cardiomyopathy (DCM) [34,35,52]. IL-17A acts on cardiac fibroblasts and induces the production of cytokines and chemokines in subtype of cardiac fibroblasts that direct immune cells such as neutrophils and inflammatory monocytes to traffic to the heart and differentiate to inflammatory subtype there [35]. Even response to Th2 can lead to cardiac damage with eosinophils as the main effector cells [53]. Similar to the response to Th17, in Th2 driven myocarditis, fibroblasts also play a major role in the chemotaxis of the effector cells, eosinophils, by the production of chemokines eotaxins [54].

Similarly, B cells are heterogeneous and have both antibody-dependent and antibody-independent functions [55]. While cardiotoxic autoantibodies have been detected in animal models of viral myocarditis, it remains unclear whether such autoantibodies are pathogenic in humans [56,57,58]. Besides recognizing specific antigen, antibodies can also regulate inflammation via Fc receptors. Moreover, antibody-independent B cell functions may be critical to the development of myocarditis and DCM. B cells are sources of abundant cytokines (such as IL-10) and growth factors (such as GM-CSF). B cells can express Toll-like receptors (TLRs) and present antigens. It is therefore essential to clarify the roles of antibody-dependent and antibody-independent B cell functions in viral and autoimmune myocarditis.

Overreactive innate immune response can contribute to cardiac inflammation as well, especially when pericarditis is also present, as was shown by IL-33 induced pericarditis [59,60]. Interestingly, IL-33 induced perimyocarditis can develop by activation of innate lymphoid (ILC) cells without contribution of adaptive immune system [60]. The ILCs have a unique profile in the heart, which only underscores the need to understand the overall role of innate immune response in myocarditis and cardiac injury.

It is important to consider sex differences in myocarditis because men are more likely than women to develop myocarditis and DCM [61,62]. Most immune cells express sex hormone receptors [63], and studies using mouse models of myocarditis with viral infection have shown increased inflammation in males and important roles for sex hormones and TLRs, especially TLR2 and TLR4, in the pathogenesis of the disease [61,64,65,66,67]. Males in both mice and human have elevated levels of CD11b+ immune cells, sera soluble suppression of tumorigenesis-2 (sST2) and Th17-type immune responses during myocarditis [52,61,68,69]. Furthermore, mouse studies of viral myocarditis have shown that testosterone increases myocardial inflammation, while estradiol reduces it [61,65,70,71,72]. Thus, in addition to defining the immune cell landscape, examining sex and age differences in viral and experimental autoimmune myocarditis animal models will be critical.

**Gaps and Opportunities in Pathogenesis of Myocarditis:**

- Further research into the role of innate and adaptive immune response in myocarditis and cardiac injury in both viral and autoimmune myocarditis is essential for the discovery of new diagnostics and treatments for myocarditis.
- Cardiac injury during the COVID-19 pandemic has revealed gaps in the knowledge of cardiac inflammation including the heterogeneity of cellular signaling, temporal sequence and regulation of inflammatory processes in cardiac tissues. New diagnostic methods including single cell sequencing linked to deep clinical phenotyping are needed to advance out understanding and identify new and potentially druggable targets.
- Lack of systemic and heart-specific immunophenotyping tools that can be deployed at the bedside are needed to understand distinct mechanism-based subgroups and develop more specific treatment strategies with acceptable risks.

## 4. Animal Models

**Figure 2:**
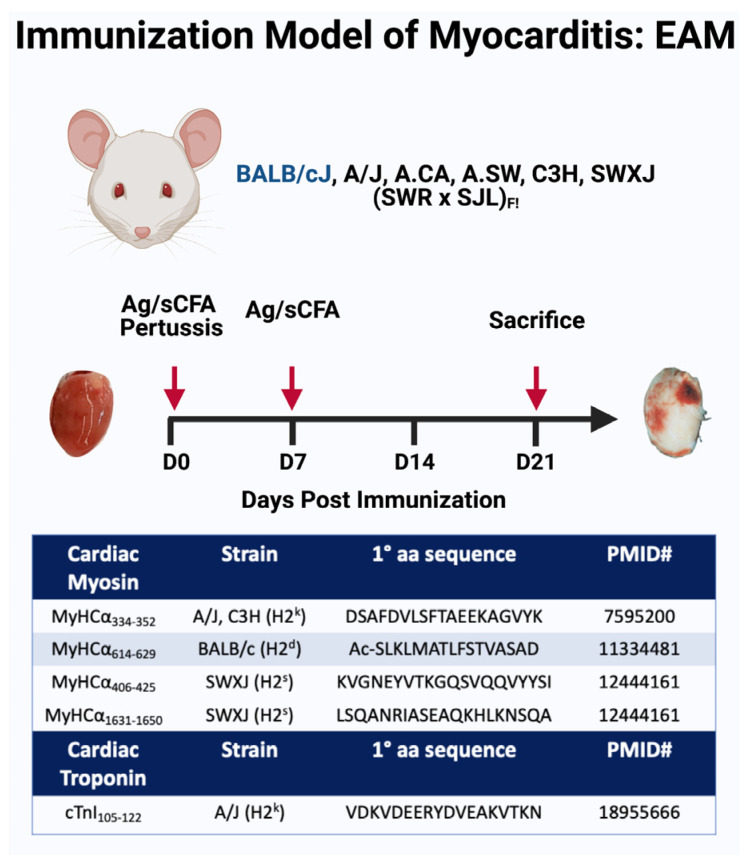
**Experimental autoimmune myocarditis murine model.** In the EAM model, cardiac myosin or the relevant peptide in Freund’s complete adjuvant is injected subcutaneously into mice on day 0 and day 7. The immune response, the histological changes, and the genetic susceptibility observed in EAM are similar to giant cell myocarditis. #: The numbers are identifying the epitope of myosin heavy chain (MYHC) used for the induction of myocarditis.

The current understanding of the pathogenesis of myocarditis comes largely from mouse models. Two Coxsackievirus B3 (CVB3) models are widely used. The first model can induce severe myocarditis and death of the infected mice within the first week, while the second model utilizes less severe heart-passaged CVB3 that allows a study of dilated cardiomyopathy phase [73,74]. Autoimmune myocarditis similar to human giant cell myocarditis can be modeled as experimental autoimmune myocarditis (EAM) [75,76]. The EAM is induced by immunization with an alpha myocyte heavy chain (αMHC) peptide emulsified in an adjuvant such as complete Freund’s adjuvant in susceptible strains of mice, such as A/J, Balb/c and SWXJ mice [33,34,46,53,77,78] (Figure 2). The EAM models all induce a Th17 response in the heart with neutrophils and inflammatory monocytes, leading to cardiac inflammation, cardiac fibrosis and dilated cardiomyopathy in susceptible mice. Troponin-induced myocarditis has also been developed [79]. Eosinophilic myocarditis mouse models as EAM in mice that overexpress IL-5 under the CD3δ promoter or mice deficient in both IL-17A and IFNγ (IL17A-/-IFNγ-/-) have been also described [80]. Recently, a spontaneous mouse mutant displays hypereosinophilia and develops eosinophilic myocarditis that results in sudden, premature death of the animals [81]. In addition, spontaneous myocarditis develops in PD-1 knockout (KO) mice on the MLR-Fas^lpr/lpr^ background [82]. Pharmacological models and genetic mouse models of myocarditis induced by ICIs have also been in development; such models are essential for understanding the pathogenesis of this rare but serious complication [83]. Despite the availability of these models, additional models reflecting the diverse etiologies, pathogenesis and outcomes of myocarditis are needed. Additional viral myocarditis models are essential to understand viral-induced cardiac injury as was clearly shown by the COVID-19 pandemic.

**Gaps and Opportunities in Animal:**

- A broader spectrum of myocarditis models of different etiologies are needed to understand emerging causes of myocarditis including COVID-19 myocarditis, ICI-induced myocarditis, and an mRNA vaccine-induced myocarditis.
- Efforts to standardize myocarditis animal models across different labs would improve the translatability of the basic research to bedside medicine.
- Higher utilization of an in vitro approach and organelles development would aid the study of some aspects of myocarditis pathogenesis.

## 5. Genetic Regulation of Myocarditis

Polymorphisms in MHC are among the strongest predisposing genetic factors in autoimmune diseases in general, and serologic and molecular analyses suggest a significant, but inconsistent correlation of DCM with MHC class II antigens, particularly HLA-DR4, DQ5, and DQ8 [84,85]. Further, specific immune response associated genes related to autoimmune myocarditis have been identified, including genes for CD45, cardiac actin, cardiac β-myosin heavy chain, cardiac troponin T, CTLA-4, ICOS, TLR-3, and interferon-induced transmembrane protein 3 (IFITM3), PD-1 [86,87,88]. Interestingly, viral myocarditis may also have genetic underpinnings. Elimination of the Coxsackievirus-Adenovirus Receptor (CAR) in adult hearts has been shown to block virus entry into the heart and to prevent contractile dysfunction. The increased expression of CAR is associated with increased incidence of both myocarditis and DCM [89]. Even more importantly, associations of acute myocarditis with familial inherited cardiomyopathies were discovered, including genes involved with arrhythmogenic right ventricular cardiomyopathy that play a role in cardiomyocytes sarcomere, nuclear membrane, Z-disc, desmosome, and ion channels, such as *DSP*, *DSG2*, *PKP2*, *TTN DYSF*, and *TNNI3* (encoding desmoplakin, desmoglein 2, plakophilin-2, titin, dysferin and troponin I type 3, respectively) [90,91,92,93,94]. These new discoveries demonstrate that genetic predisposition to all types of myocarditis should be examined further. It is essential that the consequences for the susceptibility to myocarditis and differences in immune response be examined using in vivo and in vitro models. An example of such follow-up investigation is a study examining how dystrophin affects susceptibility to Coxsackievirus [95]. Most of the available data about genetic predisposition to myocarditis come from case reports or small case series underlining the urgent need for large, prospective multi-center studies on the genetic underpinnings of myocarditis.

**Gaps and Opportunities in Genetic Regulation of Myocarditis:**

- The role of non-immune genes in susceptibility to all types of myocarditis including viral myocarditis should be further studied.
- It is essential to examine the clinical consequences for the susceptibility to myocarditis and differences in immune response using in vivo and in vitro models.
- Prospective multi-center studies on the genetic role in myocarditis susceptibility are needed to capture the impact on disease severity and long terms outcomes.
- The establishment of an international myocarditis registry to collect genetic information and link it to patients’ clinical phenotypes and outcomes might be an important and initial step toward advancing research in genetic regulation of myocarditis.

## 6. Clinical Presentation

The diversity of clinical presentations of myocarditis and variable criteria for histological and imaging diagnosis have created challenges to the identification of useful therapeutic interventions. The workshop participants identified a need to improve classification of myocarditis into specific types and incorporate the heterogeneity of the disease into diagnostic and clinical criteria. This effort would specifically aim to standardize and integrate endomyocardial biopsy, imaging, laboratory, and clinical criteria for the understanding and management of myocarditis.

The heart consists of a complex cellular network including myocytes, fibroblasts, and immune cells with molecular and functional relationships [96]. These relationships have been probed with traditional histology and immunohistology, but the insights gained from these efforts have led to few meaningful advances in myocarditis treatment. New systems of classification based on a deeper knowledge of the cellular architecture, genetics, and epigenetic modifications of single cells within health and various disease states are needed to establish a new foundation for cardiac immune homeostasis and immunopathology.

Clinical myocarditis phenotypes have established prognostic value. For example, hemodynamic compromise with cardiogenic shock, the presence of ventricular arrhythmias, high-grade heart block, female sex and lower left and right ventricular systolic function increase the risk of subsequent death or heart transplantation [97,98]. However, the features on heart biopsy histology and cardiovascular magnetic resonance imaging (MRI) that guide therapy are limited to a few uncommon conditions, such as giant cell or eosinophilic myocarditis. The presence of viral genomes and certain regulatory microRNA or cytokine patterns in heart may further refine these risks [4]. Larger studies in diverse populations with high dimensional phenotyping are needed to achieve greater functional recovery and reduce clinically meaningful adverse events.

Addressing social determinants of cardiovascular outcomes is essential to reduce the societal burden on myocarditis. The impact of social determinants of health (SDoH), including systems to identify and treat illnesses, socioeconomic position, and race, is established for traditional risk factors and outcomes in cardiovascular diseases [99]. More recently, SDoH have also been implicated in altering the patients’ immune function and inflammatory response, and these observations have driven new hypotheses regarding disparities in COVID-19 related outcomes [100]. Within the United States, the counties with the highest rates of myocarditis are in regions of the country with high rates of general risk factors for cardiovascular disease. However, minimal research has been conducted on the role of SDoH in myocarditis risk, treatment, and outcomes.

***G* aps and Opportunities in Clinical Presentation:**

- A specific and sensitive mechanism-based diagnostic criteria that refines the detection of myocarditis across the spectrum of clinical presentations is a high priority gap.
- Efforts to quantify the impact of SDoH (e.g., using PhenX Toolkit or other survey or screening tools) on cardiovascular outcomes in myocarditis outcomes are necessary.
- Validation of tools to capture patient reported outcomes and quantitate psychosocial aspects of heath are needed to assess disease burden in diverse populations.

## 7. Diagnostic Imaging for Myocarditis

Given the non-specific and variable clinical presentation of patients with myocarditis, cardiovascular imaging plays an important role in diagnostic and therapeutic decision-making. The most common imaging modalities, i.e., echocardiography, nuclear cardiology—specifically positron emission tomography (PET), and cardiovascular magnetic resonance (CMR), demonstrate differing capabilities to assess overall ventricular structure and systolic function, localize associated wall motion abnormalities, detect inflammatory tissue changes (e.g., edema, hyperactive metabolism, hyperemia, and pericardial effusion), and identify necrosis and fibrosis (Table 1 provides a succinct overview of these modalities). Because inflammation itself is a non-specific response to any myocardial injury, the clinical history and current status are important, as such contextual information allows a determination of pre-test likelihood, which in turn provides the range of the estimated post-test likelihood and eventual management decisions. Beyond diagnosis and treatment, cardiovascular imaging may provide prognostic information that better identifies which patients require closer monitoring and follow-up.

**Echocardiography** is often an initial choice of cardiac imaging to obtain an overview of ventricular size and function and plays a special role in the assessment of myocarditis because of its easy availability and ability to be employed as a bedside technique in emergency settings. Yet, in the absence of wall motion abnormalities not explained by acute ischemia, echocardiography lacks the sensitivity to detect inflammatory markers, thus limiting its clinical utility. Data are limited on specific diagnostic criteria for echocardiography in patients with suspected myocarditis; however, over the clinical course of myocarditis, echocardiography may also be used to monitor structural and functional changes in the heart. Newer methods, such as speckle tracking strain imaging, allow assessments of global longitudinal strain or global circumferential strain as indicators of left ventricular dysfunction [101]. Deformation is affected by myocardial wall properties (e.g., hypertrophy, fibrosis, infiltration, and inflammation) [102]. Differentiation of endocardial versus the epicardial strain abnormalities seen commonly in myocarditis, as well as the location of the abnormality (inferolateral and anterolateral segments are often affected in myocarditis) may help to differentiate myocarditis from ischemic heart disease [103]. Strain has been shown to have additional prognostic value above ejection fraction in patients with peripartum cardiomyopathy, which may have an inflammatory component similar to myocarditis [104]. A combined approach of routine measures of systolic function, Doppler measures of diastolic function, and new measures of myocardial strain may potentially be useful as the initial step in patients with suspected myocarditis. Additional attention to right ventricular structure and function may also play a role in identifying some myocarditis etiologies (e.g., Chagas disease) [105].

**Positron emission tomography (PET)** imaging uses injected radioactive tracers to provide quantitative information on blood flow and shows a relative augmentation of regional metabolism associated with inflammation. PET is non-invasive and not subject to sampling error as opposed to biopsy. Compared to CMR, PET’s high expense, use of radioactive substances, and lower spatial resolution limit its widespread use. Nonetheless, PET is a highly sensitive molecular imaging modality with potential for more specific identification of inflammatory cells [106]. Currently, it is being used to identify presence of active inflammation and responses to therapy in cardiac sarcoidosis [107]. Still in a preclinical phase is work identifying macrophage subtypes and their role that are associated with disease progression. The role of macrophages in inducing arrhythmia also provides another area of potential interest. Early research includes investigation into the expression of specific surface markers by macrophages and their suitability for imaging, identification of the best macrophage or receptor-related target for imaging (e.g., chemokine receptor, somatostatin receptor, folate receptor, and translocator protein), and identification of the best targeting strategy (e.g., fluorodeoxyglucose, nanoparticles, antibodies to macrophage surface antigen receptors, or small molecules) [108].

**Cardiovascular Magnetic Resonance** (CMR) imaging is seen by many as the gold standard cardiovascular imaging technique with additional unique capabilities, especially for non-invasive myocardial tissue characterization for which a variety of techniques are used, including novel techniques such as T1/T2 mapping and extracellular volume mapping [109]. The updated 2018 Lake Louise criteria for CMR assessment of myocardial inflammation [110] incorporated mapping as novel techniques, and recent studies have confirmed strong diagnostic accuracy of the updated criteria in patients with a high suspicion for acute myocarditis. CMR and specifically the presence of irreversible injury as defined by late gadolinium enhancement (LGE) also have strong prognostic value in patients with suspected myocarditis [111]. Newer techniques, such as extracellular volume fraction, have also shown promise in predicting cardiovascular events [112]. However, CMR is still hampered by a lack of experienced staff in many imaging centers and the perceived complexity of the exam itself when compared to other modalities. Additionally, more work is needed in standardizing validated CMR markers and protocols into routine clinical practice to facilitate multi-center studies and inter-hospital communication.

**Future areas for development** in all cardiovascular imaging include the potential of employing artificial intelligence (AI), specifically deep learning, for identifying novel inflammation-specific biomarkers as well as patterns of combinations thereof. Integration of clinical knowledge and pre-test probability with imaging data may improve diagnostic yield but may also be incorporated into a risk prediction model. Combining imaging methods (e.g., PET-CMR) may provide additional insights into the pathophysiology of myocarditis. All modalities have the potential to target specific cells and proteins of inflammation: echocardiography using molecular bubble contrast; PET focusing upon metabolic macrophages; and CMR utilizing nanoparticles of iron oxide [106,113]. Imaging-directed endomyocardial biopsy may improve the sampling diagnostic yield (e.g., interventional CMR methods). Further work is required to better phenotype myocarditis and establish criteria for the different infectious, autoinflammatory, rheumatologic, and oncologic-therapy-induced etiologies. Finally, to successfully translate the newer imaging methods into real-world practice, there exists the need for robust multi-center clinical trials and myocarditis patient registries in order to evaluate the impact of these diagnostic imaging procedures on clinical outcomes.

**Gaps and Opportunities in Diagnostic Imaging for Myocarditis:**

- The nonspecific presentation of myocarditis mandates improved discriminatory diagnostic testing using molecular targets for specific etiologies.
- Molecular targets of inflammation should be leveraged to improve the specificity of imaging methods.
- Integration of clinical imaging and immunological data within AI-assisted risk prediction models should be explored to improve risk assessment models.

## 8. New Diagnostic and Therapeutic Targets for Myocarditis

Systems biology and network medicine approaches are rapidly evolving to gain further mechanistic information from complex datasets generated by advanced phenotyping with high-resolution “multi-omics” screening from animal models of myocarditis and more importantly from individual myocarditis patients [114]. Application of network principles (e.g., the protein–protein interaction network or ‘interactome’) may someday address the current challenges in applying precision medicine diagnostics and therapeutics [115]. Mapping gene/protein variants and differentially expressed proteins relevant to myocarditis to create unique ‘reticulotypes’, such as individually curated clinical and molecular data, may identify optimal drug targets or facilitate the rational repurposing of approved drugs whose targets may be closely linked to pathways relevant to unique patients with myocarditis [116]. In addition, proteomics has an emerging role in the diagnosis of myocarditis. The evidence of circulating biomarkers indicating inflammation, such as c-reactive protein (CRP), ST2, myocardial injury (troponins), and autoantibodies directed against cardiac proteins, suggests that myocarditis has a broad systemic impact in affected individuals. The proteomics methodology can be applied to precisely quantify circulating proteins or identify autoantigen/antibodies in plasma or dried blood samples from self-remote blood sampling devices [117].

The emerging roles of next generation sequencing (e.g., sc-RNAseq and advanced proteomics including spatial expression) are needed to improve the ability of classification to predict outcomes and identify therapeutic targets [118]. These new tools are poised to delineate the importance of resident cardiac macrophages in myocarditis and explore the therapeutic potential of modulating their activation and effector functions. Potential areas of focus could include the influence of different cardiac resident macrophage populations on innate and adaptive immune responses, tissue repair and remodeling, pathogen clearance, and autoimmunity. Further investigations into the contributions of immune cells to basal myocardial homeostasis (independent of classical acute inflammation), and cardiac alterations that are possibly associated with immune anomalies such as that seen with age or chronic infection are warranted. It is critical to leverage unbiased technologies that identify immune cell subpopulations and utilize genetic tools that separate the function of closely related cell types or identify the evolutionary conserved immune subsets and pathways between mice and humans in various models of cardiac injury. Such approaches may facilitate the understanding of the plasticity of monocytes, dendritic cells, and macrophages in the diverse natural histories of myocarditis.

Regarding systems biology approaches to myocarditis, there are ongoing challenges in reliably generating data on individual patients’ genetic variation, gene expression and regulation, as well as environmental factors. Integrating these data with clinical information on phenotype, imaging, and lab values is essential to gain insights of comprehensive networks that are relevant to myocarditis. Scalable strategies to overcome operational logistical hurdles in data sharing, privacy protection, and data integrity/quality/reliability of patient information, as well as promotion of workforce development and team science in the clinical practice settings are desperately needed. Additionally, there is a critical need to translate such sophisticated analytical approaches to bedside clinical applications, and to demonstrate enhanced clinical value for managing and preventing patients with myocarditis.

**Figure 3:**
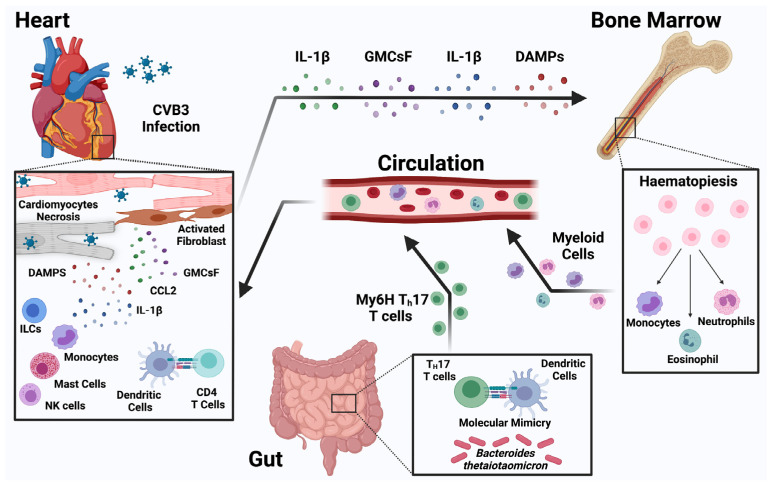
**Immune regulation of coxsackievirus B3-induced myocarditis.** Coxsackievirus B3 has the ability to infect cardiomyocytes. The innate immune system including ILCs, NK cells, macrophages, and dendritic cells in the heart is activated after the release of damage-associated molecular patterns (DAMPs). Cardiac stroma cells such as fibroblasts are also activated. Together with the immune cells, they release chemokines and cytokines that attract other immune cells from circulation. In addition, bone marrow is activated and increases hematopoiesis by the heart-derived mediators such as IL-1β, GM-CSF, and DAMPs. The gut is a possible other source of inflammatory cells such as Th17 cells that could have been primed by gut microbiota.

The COVID-19 pandemic has demonstrated that new viruses can cause myocarditis and cardiac damage with more complex pathogenesis. In addition, new mRNA vaccines will be used more broadly for other viruses, and there is an urgent need to understand the pathogenesis of the rare myocarditis side effect that they can cause. In addition, recent reports place myocarditis as a critical adverse event of using ICIs during cancer therapy. New ICIs therapies are soon to be approved or are in clinical trials. They will also likely be used in combinations with other toxin modalities and more myocarditis cases may develop as an adverse reaction. There is an urgent need to understand the role of ICIs in cardiac immune homeostasis, model the ICI-induced myocarditis in vivo, and define biomarkers of susceptibility to this cardiac injury. It was shown that in genetically susceptible individuals, elevated *Bacteroides*-specific CD4^+^ T cell and B cell responses contributed to myocarditis [119]. There is a need to further elucidate the spatiotemporal events unleashed by microbiome-driven activation of cross-reactive CD4+ T cells underlying inflammatory cardiomyopathy. It is also conceivable that manipulation of the microbiome in those affected with or at risk of developing acute myocarditis (such as patients receiving ICIs) may represent a promising strategy to dampen and/or prevent the severe cardiac inflammation observed in these patients [119] (Figure 3).

Furthermore, perturbations in autonomous elimination and replacement of dysfunctional mitochondria by a dense network of macrophages in the heart via membrane-surrounded large vesicles (“exophers”) can display activation of inflammasomes, autophagy, and metabolic dysfunction during inflammatory reactions, such as those seen during myocarditis [120,121]. Chimeric antigen receptor engineered T cells (CAR T cells) could be used to target pathologic cardiac fibroblasts that express Fibroblast Activation protein [122]. These studies showed that adoptive transfer of T cells that express a chimeric antigen receptor against fibroblast activation protein led to a significant reduction in cardiac fibrosis and restoration of function in an angiotensin II/phenylephrine induced model of cardiac injury [122]. These studies emphasize the emerging role of novel cell therapies for cardiac diseases.

**Gaps and Opportunities in New Diagnostic and Therapeutic Targets for Myocarditis:**

- Proteomic studies on archived samples of patients with myocarditis should be integrated with genetic data and cardiac imaging to deep phenotype patients with myocarditis and guide the development of new diagnostic and therapeutic targets.
- An improved understanding through model systems and patients at risk for developing of ICI-associated myocarditis are needed to dissect the pathogenesis of ICI myocarditis and design the next generation of treatment trials.

## 9. Summary

The NHLBI workshop on myocarditis highlighted existing gaps and opportunities in diagnosis, treatment, and understanding of the myocarditis pathogenesis, including sex differences in myocarditis incidence and outcomes. These recommendations form the foundation for the next step, which is the development of a new classification scheme for myocarditis that integrates current understanding of cardiac immunology. Some of these gaps became apparent due to the COVID-19 pandemic with new disorders of SARS-CoV2-induced myocarditis and post-COVID-19 mRNA vaccine myocarditis. Major conclusions from this workshop include the need to deepen our knowledge of pathogenesis of these new etiologies of myocarditis in order to develop etiology-specific diagnostic tests and treatments. Given the potential of mRNA-based technologies for preventing other infectious diseases in the future, understanding the mRNA-vaccine-induced myocarditis is of a paramount public health importance. A second conclusion is the urgent need for a better understanding of cardiac injury associated with ICIs. Given the high mortality and growing number of ICIs used for cancer therapies, it is essential to understand the susceptibility of such myocarditis and to identify biomarkers for an early diagnosis. Improved in vitro and in vivo models will help better discern the detailed roles of innate and adaptive immune responses in myocarditis. Advanced molecular tools, such as single-cell sequencing and proteomics, will allow phenotyping of immunological myocarditis subtypes. The interplay of genetic susceptibility to myocarditis has become an important new area that will require further investigation. Detailed recommendations for management are beyond the scope of this workshop; yet the translational multicenter studies utilizing archived samples of patients with myocarditis that we propose will impact management. Most importantly, this workshop concludes that clinical treatment trials that combine insights from genetics, tissue analysis at the single-cell level, and cardiac imaging to phenotype patients with myocarditis and a spectrum of inflammatory myocardial diseases are feasible today and essential to identify new diagnostic and therapeutic targets to reduce the societal burden of myocarditis.

## Figures and Tables

**Table 1:** An Overview of Imaging Modalities Used in the Evaluation of Patients Presenting with Myocarditis.

	Transthoracic Echocardiography	Positron Emission Tomography	Cardiovascular Magnetic Resonance
Ventricular volumes, myocardial mass, systolic function	+++	++	++++
Ventricular strain, myocardial mechanics	++++	++	+++
Inflammation	++	++++	++++
Fibrosis/Infiltration	++	+++	++++
Pericardium/Pericardial effusion	+++	++	++++
Alternate diagnoses of chest pain syndromes	++	+++	++++
Cost	Inexpensive	Most expensive	Increasingly affordable; cost-effective
Strengths	Portable, widely accessible in most medical institutions, rapid assessment of systolic function and wall motion abnormalities	Highly validated in inflammatory state; molecular and metabolic characterization	High spatial resolution, highly reproducible, volumetric coverage, multi-faceted tissue characterization
Limitations	Dependence on optimal acoustic window, nonspecific findings	Exposure to ionizing radiation with use of nuclear tracers, special preparation required	Specific Hardware/software requirements with fewer centers of excellence available; claustrophobia, use of gadolinium contrast

A higher number of “+” symbols indicates a higher strength or utility in the category.
